# Overview of Existing Research on Crisis-Support Training in Virtual Reality

**DOI:** 10.1192/j.eurpsy.2025.215

**Published:** 2025-08-26

**Authors:** R. Knez

**Affiliations:** 1 Skaraborg Hospital; 2 Univesity of Skövde, Skövde, Sweden

## Abstract

**Abstract:**

This presentation will provide an overview of existing research on crisis-support training in virtual reality, highlighting both the opportunities and challenges of utilizing this training method. It will review current research trends in the field, identify gaps in knowledge, and propose directions for future research initiatives. Studies on crisis-support training that utilize a virtual reality training approach for professionals working with children and adolescents will also be presented.

**Disclosure of Interest:**

None Declared

